# Antifungal Immunological Defenses in Newborns

**DOI:** 10.3389/fimmu.2017.00281

**Published:** 2017-03-15

**Authors:** Christina Michalski, Bernard Kan, Pascal M. Lavoie

**Affiliations:** ^1^British Columbia Children’s Hospital Research Institute, Vancouver, BC, Canada; ^2^Department of Medicine, University of British Columbia, Vancouver, BC, Canada; ^3^Department of Pediatrics, University of British Columbia, Vancouver, BC, Canada

**Keywords:** neonate, immunology, fungus, *Candida*, humans, infection, prematurity

## Abstract

Newborns are prone to fungal infections, largely due to *Candida* species. The immunological basis for this vulnerability is not yet fully understood. However, useful insights can be gained from the knowledge of the maturation of immune pathways during ontogeny, particularly when placed in context with how rare genetic mutations in humans predispose to fungal diseases. In this article, we review these most current data on immune functions in human newborns, highlighting pathways most relevant to the response to *Candida*. While discussing these data, we propose a framework of why deficiencies in these pathways make newborns particularly vulnerable to this opportunistic pathogen.

## Introduction

Fungi are present everywhere in the environment, including in water, on solid surfaces, on our skin, and gastrointestinal tract. Taxonomists estimate the existence of 1.5 to over 5 million fungal species, although only a small minority (<300 species) causes diseases in humans (1). Despite their ubiquitous presence, fungi rarely become invasive in healthy adults due to multiple levels of immune defenses. In contrast, fungal infections are common in newborns and can be particularly invasive in those born very prematurely (2, 3).

**Figure 1:**
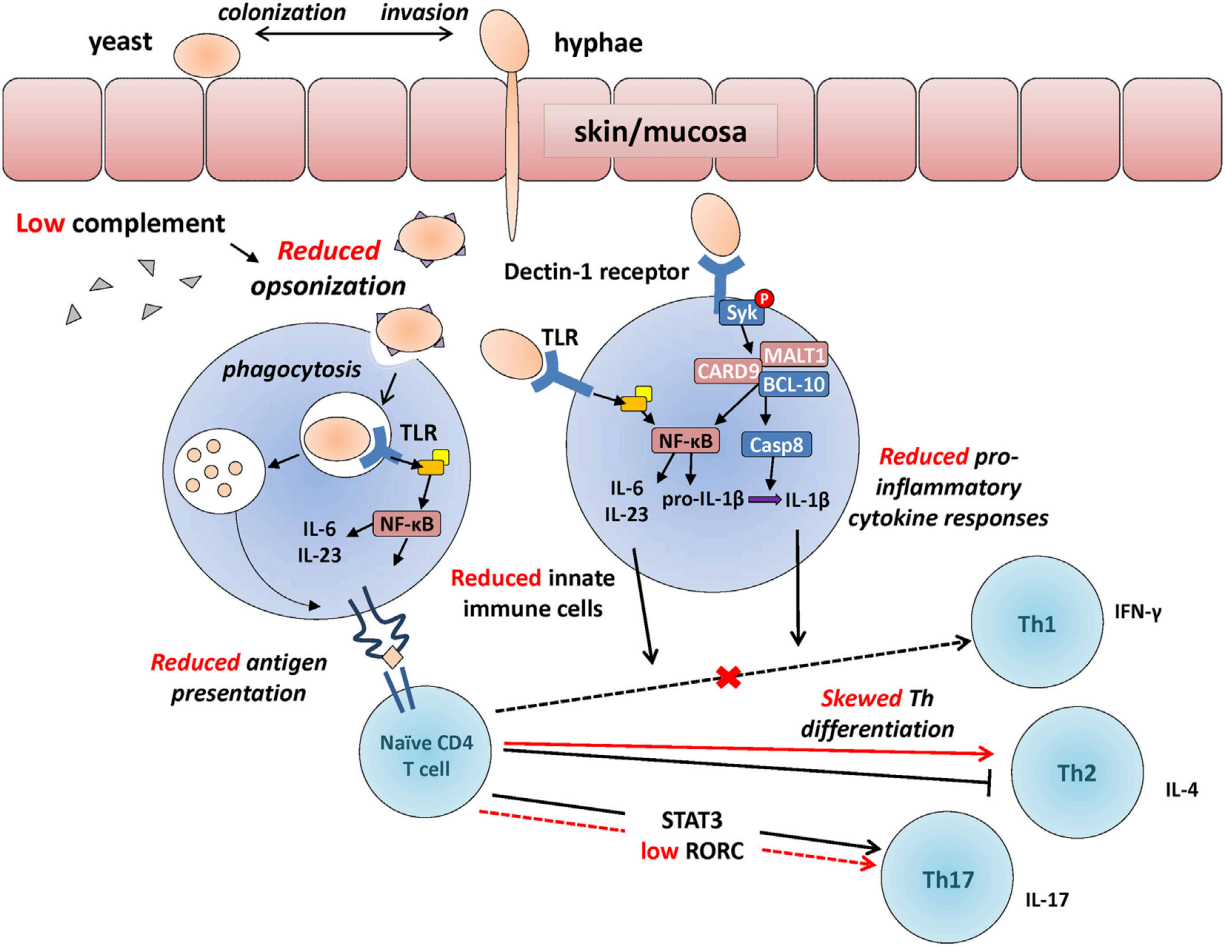
**Developmental changes in the immunological response to *Candida***. Developmental differences in some of the main immunological events involved in the recognition of *Candida*, between adults (in black) and neonates (in red, cross also indicates a reduction in neonates compared to adults). *Candida* colonizes the skin or mucosa as yeast but often invades in hyphae form allowing to penetrate through epithelial barriers more efficiently. Upon innate immune detection of *Candida* through pattern recognition receptors [e.g., toll-like receptors (TLRs); Dectin-1 receptor], microorganisms get opsonized (reduced in preterm neonates), facilitating phagocytosis and resulting in the production of pro-inflammatory cytokines (also reduced in neonates) through Syk and NFκB-mediated intracellular signaling. Internalized *Candida* antigens are presented (reduced in neonates) to naïve CD4 T cells, resulting in their differentiation into Th1/Th17 effector cells (skewed toward T helper 2 in neonates). Whereas deficiencies in innate functions (e.g., MALT-1/CARD9) can lead to invasive candidemia, selective deficiencies in adaptive functions (e.g., IL-17 responses) most often lead to chronic mucocutaneous infections.

A number of studies have investigated the functional characteristics of newborn immune cells [reviewed in Ref. (4, 5)]. The immune system is composed of two main arms involved in the recognition of fungi. Developmental changes in some of the main immune pathways involved in responses against Candida are illustrated in Figure 1. During gestation, innate immune cells are skewed toward anti-inflammatory responses. Adaptive immune cells also lack immunological memory from prior exposure to antigens and are skewed toward a T helper 2 (Th2) effector profile. These changes are essential to prevent allogeneic maternal rejection and during the establishment of tolerance toward self-antigens. Moreover, the expansion and maturation of immune cells is incomplete in infants born very prematurely, which further increases their vulnerability to infections (4, 5). These functional limitations are also affected by pregnancy complications, which can be linked to a premature birth (6, 7).

While our knowledge of the maturation of immune pathways in human newborns has greatly progressed recently, few of these studies have focused on fungi as model organisms. Therefore, our understanding of the immunological basis for the increased susceptibility of the neonatal immune system to fungi remains limited. Nonetheless, insights can be gained from rare genetic mutations predisposing to localized or invasive *Candida* infections in humans. These data have been recently covered by other experts (8, 9). The clinical presentation, risk factors, and treatment of neonatal *Candida* infections have also been reviewed recently (10, 11). This review discusses recent data underlying the immunological basis for newborns’ increased susceptibility to *Candida* infections.

## Neonatal *Candida* Infections

In newborns, *Candida* is responsible for the common oral thrush and rash in skin folds and in the diaper area. Before the advent of modern sanitary measures and topical antifungal treatments, infants died from dehydration due to severe oral mucocandidiasis (12). Nowadays, invasive infections are rare with the exception of infants born very premature, those who require prolonged indwelling medical devices, or in cases of a primary immunodeficiency (8, 13).

Once invasion occurs, the mortality from *Candida* infections in newborns is high, and so is the associated morbidity: up to two-thirds of those who survive will suffer long-term impairments (14). Similarly, fungemia due to other genera such as *Malassezia* (15), Aspergillosis (16), and Zygomycosis (17) also carry a high mortality, though these infections are more rare. Dermatophytes infrequently cause skin infections in young infants.

At birth, neonates generally have a low fungal burden (18–20); however, colonization occurs in a majority of neonates through both vertical (mother-to-child) and horizontal (nosocomial) transmission (20–28). Most invasive *Candida* infections occur between the second and sixth week postnatal age (29, 30) owing to the timing of colonization. *Candida albicans* is the most frequently isolated *Candida* species, but other species, particularly *Candida parapsilosis*, but also *Candida tropicalis, Candida glaberata*, and *Candida kruzei* are becoming more prevalent (11, 31–33). Interestingly, major variations have been reported in the incidence and species distribution of *Candida* infection among neonatal intensive care units across the world (3, 34, 35). For example, in North America and Europe, invasive disease almost exclusively occurs in infants of birth weight less than 1,000 g (2, 11, 36, 37), whereas up to 15% of infants born below 33 weeks in neonatal center in Shanghai were diagnosed with a systemic fungal infection (38). These variations may be due to racial differences in immune phenotypes, although this has not been formally examined in the context of *Candida* infections. On the other hand, these differences in epidemiology are more likely due to geographical variations in infection control measures and in the use of broad spectrum antibiotics.

## Innate Immune Responses

The innate immune system is the first-line of immune defenses responsible for signaling the presence of microorganisms and riding our body from an invasion through opsonization (i.e., targeted labeling), cell-to-organism killing, and phagocytosis. The epithelial layers (skin and mucosa) are the first line of defense of the innate immune system against a fungal invasion (39, 40). Highly premature infants lack *vernix caseosa*, which is a natural substance composed of antimicrobial sebum, covering the skin of term newborns (41, 42). This lack of *vernix caseosa* may increase fungal invasion by affecting the balance between the infant’s bacterial and fungal flora (42). However, this contention, at this point, remains speculative and requires further study.

### Antimicrobial Peptides

Antimicrobial peptides are a major component of innate immune defenses. These peptides generally show reduced levels in those born prematurely [reviewed in Ref. (43)]. Levels of α-defensin have been correlated with the presence of mannan in bronchoalveolar lavage fluids from preterm neonates, indicating a role in controlling fungal growth at mucosal surfaces (44). Complement proteins are another major component, consisting of at least 20 interdependent components that are deposited on the surface of pathogens, resulting in phagocytosis *via* opsonization, killing *via* pore-formation, and activation of inflammatory cytokine responses. In mice, deficiencies in complement proteins, particularly C3, result in an inability to clear infections due to *C. albicans* and *Candida glabrata* (45). Human C5a also appears important for induction of inflammatory cytokine responses to *C. albicans* (46). In one case, a child with a hereditary C3 deficiency was unable to opsonize this microorganism and normal function was restored with C3 supplementation (47); deficiencies in factor H and factor I have also been shown to negatively affect killing of *C. albicans* (48). Production of complement proteins is detectable early on in the fetus during ontogeny (49) and increases in a gestational age-dependent manner until the term of gestation and even after birth (50, 51). Therefore, it is possible that relative complement deficiencies in newborns may increase susceptibility to invasive *Candida* infections, but to a relatively minor extent.

### Phagocytes

Innate immune cells such as neutrophils, macrophages/monocytes, and dendritic cells play important roles in preventing a fungal invasion [reviewed in Ref. (52)]. In mice, depletion of neutrophils increased susceptibility to cutaneous *Candida* infections (53) and also increased the risk of lethal invasion following experimental mucosal damage (54). Humans with genetic defects that impair neutrophil functions, such as the autosomal recessive myeloperoxidase deficiency, are at greatly increased risk of systemic Candidiasis, suggesting an important role for neutrophils and other phagocytes (55). Neutrophil extracellular traps (NETs) facilitate killing of *C. albicans*, although their functional importance against this pathogen is debated (55–57). NET formation upon *Candida* exposure is operational in newborns and, therefore, a neonatal deficiency in NET does not explain their susceptibility to infections (58). On the other hand, neutropenia due to a central, bone marrow cause, severely predisposes to systemic candidiasis in adults (59). In the fetus, bone marrow production of hematopoietic cells rapidly increases after the 20th week of gestation (4, 5). Consequently, lower neutrophil and monocyte cell counts are observed in extremely low gestation preterm infants, which may play a more important role in increasing the risk of systemic infections in these infants (60, 61). Interestingly, neutropenia is often not observed during *Candida* sepsis in preterm neonates, in contrast to Gram-negative bacteria, which may indicate a more limited role for these cells once invasive infection has occurred (62).

In addition to a relatively limited neutrophil cell count, some studies have reported reduced neutrophil function in very preterm neonates. In a whole blood assay, reduced migration and phagocytosis of *C. guillermondii* was observed in very low birth weight (<1,500 g) infants compared to term neonates and adults (63). However, a recent study reported no difference in phagocytosis and oxidative burst between age groups (64). In general, phagocytosis functions are relatively preserved in very preterm neonates (65–67). These differences in findings may be due to differences in the assay or strain of *Candida* that have been used between these studies. More functional *in vitro* studies are required using *Candida* in order to help resolve these findings.

Monocytes/macrophages also appear to play an important role in preventing a *Candida* invasion based on mouse studies (52). Monocytes rely primarily on non-opsonic phagocytosis *via* Dectin-1 and Dectin-2 (68). To the best of our knowledge, the response of macrophages (or monocytes) to *Candida* has not been studied in infants born prematurely.

### Pathogen Recognition

The lipid bilayer of *Candida* is surrounded by chitin, an inner cell wall component made of polysaccharides (β,1-3 glucan, β,1-6 glucan) and an outer cell wall composed of N-linked glycoproteins coated with mannan polymers (69). *C. albicans* can transform between yeasts and hyphae based on the environmental conditions (70). These two forms have different virulence and elicit different immune responses due to structural changes in their cell wall (70, 71). Immune cells recognize the presence of pathogens through innate receptors called pattern recognition receptors (PRRs). PRR can be free circulating in body fluids (e.g., pentraxin, collectins, or ficollins) or cell associated. Cell-associated PRRs include toll-like receptor (TLR), C-type lectin receptors (CLRs), and the intracellular (cytoplasmic) NOD-like receptors (NLRs) and RIG-I-like receptors (RIRs). Several PRR are involved in the immune recognition of *Candida*, including TLR2, TLR4, TLR6, and CLRs and in the recognition of *Candida* DNA (e.g., TLR3 and TLR9) (72–74). Recognition of fungi by multiple PRRs triggers a cascade of immune activation events including the production of cytokines, reactive oxygen species, and the activation of phagocytosis. These multiple levels of immune recognition enhance immune protection in healthy individuals.

In newborns, PRR functionality develops early in the third trimester of gestation, beginning with endosomal/cytoplasmic PRR around 20–24 weeks, followed by extracellular PRR until about 33 weeks of gestation when the PRR functionality compares to that of full-term infants (7, 75, 76) [reviewed in Ref. (4, 5)]. These maturational changes are likely to play an important role in preterm infants’ increased vulnerability to infections. Indeed, cytokine responses (TNF-α, IL-6, IL-1β, and IFN-γ) are decreased and skewed toward an anti-inflammatory phenotype early in gestation (5). Reduced cytokine responses have been linked to reduced downstream signaling, in part due to decreased expression of the main MyD88 signaling molecule, as well as a gestational age-dependent reduction in phosphorylation of p38 and ERK1/2 (65, 77–81). The *S*-type lectin receptor Galectin-3 is expressed on neutrophils, monocytes, macrophages, endothelial cells, and epithelial cells, can be secreted, and confers protection in *Candida* infection leaving galectin-3-deficient mice more susceptible to *Candida* infection (82, 83). However, conflicting results have been published regarding whether Galectin-3 levels are higher (84) or lower (83) in cord vs. adult blood and whether the levels increase (85), decrease (86), or remain constant (83) with decreasing prematurity.

### Dectin-1 Receptor

Dectin-1 is a CLR and main extracellular PRR mediating the recognition of β-glucan in the *Candida* cell wall. Reduced Dectin-1 receptor function naturally occurs in ~1% of the general population due to a genetic polymorphism that introduces a stop-codon in the *CLEC7A* gene encoding this receptor. Humans with this polymorphism may display a marginally increased susceptibility to cutaneous fungal infections (87). However, these infections are generally mild due to a high degree of functional redundancy with other PRRs such as Dectin-2 (88, 89). Upon recognition of β-glucan at the surface of *Candida*, a phagocytic synapse containing Dectin-1, active Src, and Syk kinases is formed (90). The intracellular signaling molecule Syk becomes phosphorylated, resulting in the cytosolic colocalization of the signalosome complex composed of CARD9, MALT1, and Bcl-10 (see Figure 1). Assembly of this protein complex leads to two main sequences of events: (1) nuclear translocation of the transcription factor and main inflammatory regulator NF-κB, which then leads to induction of pro-inflammatory cytokine gene transcription (91) and (2) activation of the caspase-8 enzyme, which cleaves pro-IL-1β into its mature, secreted IL-1β form. Because of the central importance of the signalosome complex in antifungal immune defenses, a deficiency in CARD9 or MALT1 results in a marked increased risk for invasive fungal infections in humans (8, 92, 93). The function of Dectin-1 signaling has not been studied in premature newborns, requiring further studies to understand how this pathway may increase their susceptibility to fungal infections.

## Adaptive Immune Responses

Adaptive immune responses, mediated through dendritic cells, B and T lymphocytes, are essential to limit a *Candida* invasion. Following penetration of *C. albicans* through epithelial surfaces, dendritic cells become activated through PRR, resulting in their uptake and presentation of antigen fragments to CD4 T lymphocytes (also called “helper lymphocytes”). CD4 T cells producing the cytokine interleukin-17 (termed Th17 cells) are particularly important for controlling the proliferation of *Candida*, as evidenced by increased chronic mucocutaneous candidiasis in humans with genetic mutations in cytokines (e.g., IL-17A, IL-17F), receptors (e.g., IL-12β1R), or transcription factors (e.g., RORC, GATA2, STAT1, APS-1, and ACT1) along these pathways (94). In newborns, T cells are largely naïve and display reduced activity against microbial antigens as they have not been exposed during gestation (95). Moreover, neonatal CD4 T cells are intrinsically less able to differentiate into Th17 cells due to reduced expression of the transcription factor RORC (96). Adults with genetic mutations impairing RORC or STAT3 function have increased susceptibility to chronic mucocutaneous candidiasis due to diminished Th17 function (97, 98). STAT3 phosphorylation occurs in neonatal T cells although whether reduced expression may limit Th17 differentiation in this age group is unclear (99). Neonatal T lymphocytes also have a reduced ability to differentiate into interferon-γ-producing CD4 lymphocytes (5), which play a role protecting against fungi, through the activation of other cellular immune components (e.g., phagocytes) (9).

The role of innate immune cells in promoting the development of Th17 responses has been studied in newborns. In term newborns, antigen-presenting cells produce high levels of Th17-polarizing cytokines (i.e., IL-1β and IL-23) (100). However, the production of these cytokines and antigen presentation are profoundly reduced in dendritic cells and monocytes of preterm infants below 29 weeks of gestation (7, 101), which may further contribute to their susceptibility for invasive fungal infections. Other T lymphocyte subsets such as γδ T cells develop early during fetal life and are able to produce interleukin-17, naturally, in an innate-like manner in the absence of effector differentiation (102). In mice, these cells are an important source of interleukin-17 (103). However, their functional role in preventing fungal invasions in premature newborns remains to be determined.

## Limitations of *In Vitro* Studies

An important limitation of studies investigating fungal immune responses by human primary immune cells *in vitro* is that this situation may not reflect the complex life cycle of this microorganism during an infection *in vivo*. For example, heat-killed *C. albicans*, which is commonly used as a model in *in vitro* assays, exposes more β-glucan on its surface than live yeast (104). Dectin-1 specifically recognizes β-glucan structures in the cell wall of yeast, but not hyphae forms of *C. albicans*, where the β-glucan is less accessible to immune cells. As such, filamentous growth of *C. albicans* is not recognizable by Dectin-1, resulting in deficiency of ROS production and a reduction in Th17 differentiation of T cells (104, 105). However, *Candida* hyphae, but not yeast, induce a strong immune response in macrophages (71). Hyphae can specifically activate the NLRP3 inflammasome, which is important for production of IL-1β (106). Moreover, these changes can be strain specific (107). These limitations may significantly restrain interpretation of data.

Likewise, experimental conditions influence the interaction between immune cells and *Candida*. Sasse and colleagues showed that neutrophils can phagocytose yeast *Candida* in a suspension (3D-setting) but fail to phagocytose opaque cells on a surface, 2D-setting (i.e., glass slide) (108). Moreover, it has been suggested that yeast are important for colonization and hyphae are responsible for invasion and that the switching between the two forms itself is responsible for activation of the immune system [reviewed in Ref. (109, 110)]. Unfortunately, this is not accounted for in most *in vitro* studies as live fungal pathogens are rarely used. To mitigate these problems, animal models have been developed (111) [reviewed in Ref. (112)]. However, it is important to remember that mice are not a natural host for *Candida* and that considerable differences in immune functions across species warrant validation in humans (113).

## Enhancing Neonatal Antifungal Immune Defenses

Basic science research findings need to be translated into clinical practice. Systemic antifungal drugs reduce the incidence of colonization and invasive fungal infections (114). However, the applicability of these approaches is somewhat limited by concerns of increasing antimicrobial resistance (115). Also, the microbial flora of preterm infants differs considerably from adults, or term infants, suggesting a role for a bacterial dysbiosis in promoting Candidemia in preterm neonates (116). Indeed, one study showed high fungal diversity in stool samples from very low-birth weight infants (117). In light of these findings, altering the gastrointestinal flora of preterm infants through the use of probiotics may represent a more viable approach to reduce the risk of invasive infections in the neonatal intensive care unit (118). Reciprocally, a better understanding of the immune response to *Candida* in newborns could help design vaccine interventions (119).

More research is required to understand how immune responses can be modulated specifically in the very preterm infant. Innate immune training using ultra-low exposure to β-glucan enhances responses to *Candida in vitro* (120). In support of the application of this concept to newborns, TLR and Dectin-1 co-stimulation induced strong Th1-polarizing conditions in neonatal dendritic cells *in vitro* (121). However, without a clear knowledge of whether these pathways are functional in premature neonates, the applicability of this strategy in preventing systemic infection in the youngest age group of neonates remains speculative. Research in this area has been traditionally hard to pursue due to obvious ethical and logistical factors (4, 122). Indeed, blood volumes are extremely limited in these small infants even using non-invasive sources such as the placenta. The challenge of enrolling a large enough number of premature neonates into clinical trials is also a major limitation (3). In the absence of interventional studies, basic science research remains crucial to lay the foundation for more evidence-based medicine in our approach to neonatal fungal infections.

## Author Contributions

CM, BK, and PL conceived, wrote, and edited this manuscript.

## Conflict of Interest Statement

The authors declare that the research was conducted in the absence of any commercial or financial relationships that could be construed as a potential conflict of interest.
